# Supplementary material for: Learning curve and surgical outcome of robotic assisted colorectal surgery with ERAS program
Source: Sci Rep. 2022 Nov 29;12:20566. doi: 10.1038/s41598-022-24665-w (PMC9709162; doi:10.1038/s41598-022-24665-w)
Supplement: Supplementary file 2 — Supplementary Information 2. [file 41598_2022_24665_MOESM2_ESM.zip › RAL Raw data-Ñ[▒K/109/HS_ñw╝╨░O▒Kñσ.pdf]

正式摘要(電子簽章：000000223999387)<br>W52 / W52 ☐英文標題(注意:無英文姓名-請補病人基本檔!)	☐【讀取】憑證	

臺中榮民總醫院

出院病歷摘要

轉入號 : 轉出號 :

[redacted]

(8)流水編號	
(9)入院日期	110/08/24 (2021) 直腸外科 W52 - 143
(10)轉科日期	
(11)出院日期	110/08/31 (2021) 住院天數計 7 日

診斷

- 入院 :Colorectal cancer with distal metastasis
- 出院 :Colorectal cancer with distal metastasis

主訴

- Bloody stool for 2-3 weeks

病史

- This is a 56 year-old man, had history of DVT, protein S deficiency disease. Denied any systemic disease before. This time, he had bloody stool noted for recent 2 -3 weeks. According to the patient, he had bloody stool for recent 2-3 weeks, due to those, he went initially to LMD for help, however, his condition didn't improved after medication, therefore, he was transfer to 台中H. for help. Colonscope was arrange, however study couldn't complete due to obstruction. Due to those, he was advised to came to our CRS OPD for help. Under impression of S-colon tumor, he was admitted for further sugical intervension.

體檢發現

- 1. Imaging date: 2021/08/20 (Y/M/D) Imaging modality: (v) CT scan / ( ) MRI Imaging protocol: Range: whole abdomen Slice thickness: 5 mm Pre-contrast, axial imaging Post-contrast, axial imaging and coronal reformation 2. Tumor location and size Location: ( ) Cecum / ( ) Ascending / ( ) Hepatic flexure / ( ) Transverse / ( ) Splenic flexure / ( ) Descending / (v) Sigmoid / ( ) Rectum / ( ) Other: [ ] Size: ( ) Non-measurable (v) Measurable: [5] cm (greatest dimension) 3. Tumor invasion ( ) Not assessable ( ) No or Equivocal (v) Yes: ( ) Colonic or rectal wall (v) Invades non-peritonealized pericolonic or perirectal tissues ( ) Invades visceral peritoneum or adjacent organs: [ ] 4. Reg
    - General appearance :
        - easy looking , conscious clear , without cardiopulmonary distress
        - Vital signs自動帶入date 體溫(BT) 脈搏(P) 呼吸(R) 血壓(BP) 身高(BH) 體重(BW) BMI
20210824 36.8℃ 81次/分 19次/分 120/69mmHg 173.9cm 60kg 19.8
        - Vital signs: B.P. 120/69 mmHg , P.R. 81 /min , R.R. 19 /min , B.T. 36.8 ℃
    - HEENT :
        - Eyes: no anemic conjunctiva, no icteric sclera, normal EOM
        - ENT: no otorrhea, no rhinorrhea, no erythematous mucosa, no injected tonsil, no deformity
    - Neck: supple, freely movable, trachea midline,no deformity, no engorged jugular vein , no palpable thyroid or lymph node enlargement
    - Chest:
        - Inspection: Grossly normal, symmetric and fully expansion, no accessory muscle used, no operation scar, no spider angiomata, no gynecomastia
        - Palpation: no palpable mass or nodule, no subcutaneous emphysema, no tenderness
        - Ausculation: clear breathing sound
    - Heart:
        - Percussion: normal heart size
        - Ausculation: regular heart beat, normal S1, S2, no gallop, no murmur
    - Abdomen:
        - Inspection: in
            - flat contour, symetrically, no scar, no stria, no dilated veins, no rashes

-
  - Ausculation: normoactive bowel sound, no abdominal bruits
  - palpation: soft, no muscle guarding or rigidity, no local tenderness, no rebounding pain, no palpable mass, no palpable pulsation
  - Percussion: no shifting dulllness, no hepatomegaly, no splenomagaly
-

Peripheral pulsation :	CA	BA	RA	FA	PA	PTA	DPA
Right side :	++	++	++	++	++	++	++/+++
Left side :	++	++	++	++	++	++	++/+++

- Genito-anus: no tenderness or palpable mass lesion or rectal shelf, normal external genitalia, normal anal tone, brown stool

CRS CONDITION

- CEA: 50.20 ng/ml
-
  - Lung

手術日期及方法

- 手術日期及方法20210826 Robot Assisted Colectomy(L)

住院治療經過

- This is a 56 year-old man, was admitted due to colorectal cancer with distal metastasis.
  After admission, robotic assist colectomy was performed on 08/26.
  After the surgery, diet was gradually switch from liquid diet to soft diet. Wound was clean without abnormal discharge. Under stable condition, he was discharge today with medication, and advised OPD follow up

合併症

一般檢查

- 累積報告 :

DATE	TIME	ALB	TP	BIL.T	BIL.D	ALKP	AST	ALT	LDH	AMY	LIPASE	NH3	Ferritin	UA
1100824	1600	4.5		0.7	0.2	176	41	46	342					
1100826	1311	3.6		0.7	0.2		34	34						

DATE	TIME	WBC	RBC	HGB	HCT	MCV	PLT	BLST	NEUT	BAND	SEG	LYM	MON	EOS	BAS
1100824	1600	7200	3.90	11.9	35.9	92.1	151		81.7			9.3	6.0	2.6	0.4
1100826	1311	7230		10.2	29.9	88.7	105		92.4			5.4	1.8	0.3	0.1

DATE	TIME	NA	K	CL	BUN	CREAT	eGFR	CA	Mg	P	CRP	ESR	PCT	D-Dimer	LACTATE
1100824	1600	140	3.8		14	1.25		9.2							
1100825	1051								2.2	2.8					
1100826	1311	138	3.6		16	1.09	74.38	8.3							

DATE	TIME	GLUCOSE	HbA1C	CHOL	TG	HDL	LDL	KETONE	CK	CKMB	TRO-T	PT-P	APTT-P	NT-pro BNP
1100824	1600	97										13.1	34.3	
1100825	1050											13.0	32.4	
1100825	1051			171	98									

特殊檢查

病理報告

- Report pending

其它

出院時情況

- 3.改門診治療

出院指示

-

藥名	劑量	劑量單位	用法	頻次	數量	數量單位	現況	天數	單位
(口)Takepron OD tab 30mg	1	TAB	PO	QDAC	7	TAB	TH	7	天
Mag. Oxide tab	2	TAB	PO	TID	42	TAB	TH	7	天
10mg Xarelto tab	1	TAB	PO	QDCC	7	TAB	TH	7	天
Ultracet tab	1	TAB	PO	Q6H	20	TAB	TH	5	天
Through tab	1	TAB	PO	QN	7	TAB	TH	7	天

- | Hirudoid Cream 40gm | | TUB | TOP | BID | 0 | TUB | TH | 1 | 瓶 |

出院計劃

主治醫師 2062F 蔣鋒帆 / 住院醫師 2365H 蘇晏申

正式摘要**(電子簽章：000000223999387)**

W52 / W52

doctorRank:B
